# Influential role of lean soft tissue in the association between training volume and bone mineral density among male adolescent practitioners of impact-loading sports: ABCD Growth study

**DOI:** 10.1186/s12887-020-02402-4

**Published:** 2020-10-28

**Authors:** Pedro Henrique Narciso, André Oliveira Werneck, Rafael Luiz-de-Marco, Yuri da Silva Ventura Faustino-da-Silva, Santiago Maillane-Vanegas, Ricardo Ribeiro Agostinete, Rômulo Araújo Fernandes

**Affiliations:** 1 Department of Physical Education, Sao Paulo State University (UNESP), Roberto Simonsen Avenue, 305. Educational Center, SP CEP: 19060-900 Presidente Prudente, Brazil; 2 School of Public Health, University of Sao Paulo (USP), Sao Paulo, Brazil; 3 Department of Physical Education, Post-Graduation Program in Movement Sciences, Sao Paulo State University (UNESP), Presidente Prudente, Brazil; 4 Department of Physical Therapy, Post-Graduation Program in Physical Therapy, Sao Paulo State University (UNESP), Presidente Prudente, Brazil

**Keywords:** bone tissue, body composition, muscle mass

## Abstract

**Background:**

Training volume is associated with direct and indirect pathways of bone adaptations. In addition, training volume is a training variable associated with lean soft tissue (LST), which has been shown to be an important predictor of areal bone mineral density (aBMD). Thus, the aim of this study is to investigate the influential role of lean soft tissue (LST) in the association between training volume and aBMD in male adolescent athletes.

**Methods:**

This cross-sectional study was composed of 299 male adolescent athletes, mean age 14.1 (1.8) years, from 9 different weight-bearing modalities. The Ethical Board approved the investigation. The adolescents reported the number of days per week they trained and the time spent training and, from this, the training volume (h/wk) was estimated. The LST and aBMD were assessed by dual-energy x-ray absorptiometry. Somatic maturation was estimated by the peak of height velocity. Mediation analysis was performed to investigate the role of LST in the association between training volume and aBMD. Level of significance was set at *p* < 0.05.

**Results:**

LST partially explained the association between training volume and aBMD in all body segments: upper limbs (58.37%; β = 0.00142), lower limbs (28.35%; β = 0.00156), spine (33.80%; β = 0.00124), and whole body (41.82%, β = 0.00131). There was no direct effect of training volume on aBMD in upper limbs (CI -0.00085 to 0.00287). Conclusion: The association between training volume and aBMD is influenced by LST in different body segments, mainly upper limbs, demonstrating that interventions aiming to enhance aBMD should also consider LST as an important variable to be managed.

## Background

Adolescence is the period in which the peak of bone mineral density (BMD) accrual occurs [1] and, hence, this period of life is crucial for preventing osteoporosis later in life [2–4]. A widely known key to optimize these gains during adolescence is involvement in physical exercise programs that present impact-loading [3, 4], which is mainly manifested through sports participation [5, 6]. In this sense, adolescents engaged in sports with high-impact (i.e. basketball, volleyball, and gymnastics) and/or odd-impact (i.e. soccer, tennis, and martial arts) may benefit from this impact-loading and present better outcomes related to bone health when compared to practitioners of low-impact sport modalities (i.e. swimming) [7, 8].

There are two mechanisms that mainly explain the positive relationship between impact-loading sports and bone outcomes [4]. First, sports participation stimulates bone remodeling through the mechanical impact generated by ground-reaction force (jumping actions, sprinting, starts, stops, running, and contact with opponent and ball), which causes transverse and torsional loads on the skeleton [3]. Second, sports participation stimulates other tissues, such as lean soft tissue (LST), which is associated with higher areal bone mineral density (aBMD), not only through the increased body mass (consequently, increased mechanical loads) [3], but also through muscular contractions [9]. This mechanism stimulates the bones to adapt (raising the recruitment of bone cells [i.e. osteocytes, osteoblasts, and osteoclasts]) to support the stress generated by the muscular action [10].

In this way, many studies have sought to understand the effects of different sport training variables on bone health, such as training load and training volume [11, 12]. In fact, the weekly training volume in some sports might be beneficial to body composition (increased lean soft tissue) [13] and bone formation in different segments [13–17]. Varley et al. [11] showed that increased training volume of male soccer players led to positive bone adaptations in bone density. Other studies have also demonstrated that athletes exposed to higher weekly training hours demonstrated higher bone outcomes [13, 14]. For these reasons, training volume has gained the attention of researchers.

In addition to the well-known important role of LST in bone [18–20], it also works as a mediator of several associations such as physical activity and muscular fitness [21, 22] and aBMD. However, the same cannot be said when analyzing the association of bone outcomes with volume of training. Although there are hypotheses and speculation about how training volume affects aBMD in different body segments [11, 15–17], to our knowledge, no studies have investigated the mediating role of LST, especially using proper statistical methods to isolate the indirect effect (i.e. mediation models) [23], in the association between training volume and aBMD in different segments with a large sample of male adolescent athletes. Thus, the aim of this study was to identify the influential role of body LST in the association between training volume and aBMD in different body segments in male adolescents engaged in different impact sports.

## Methods

### Sample

This cross-sectional study is part of the Analysis of Behaviors of Children During Growth – ABCD Growth study, from the Laboratory of InVestigation in Exercise (LIVE), which was carried out in the city of Presidente Prudente, State of São Paulo, Brazil, from October 2013 to May 2018. This study was composed of male adolescents aged between 10 and 18 years. To participate in the study, informed consent was obtained in writing from all individual participants and their parents or legal guardians. The Ethical Board of the Sao Paulo State University (UNESP) (process number: 1.677.938/2016 and process number 02891112.6.0000.5402) approved the investigation.

The study was composed of a convenience sample. Firstly, the Department of Sports of Presidente Prudente city provided a list of sports clubs (basketball, soccer, volleyball, karate, judo, kung-fu, baseball, and track and field) with athletes that met the age group targeted by the ABCD Growth Study. Then, another two sports from private sports clubs were included in the study (tennis and judo), totaling 9 sports. After that, adolescents were contacted in the sports clubs after authorization provided by parents/legal guardians and coaches. The final sample (*N* = 299) comprised adolescents who were engaged in different organized sports that include high-impact and odd-impact activities, as described by previous studies [3, 8, 24], and are positively associated with bone mineral density [3] (basketball [*n* = 63], soccer [*n* = 100], volleyball [*n* = 2], karate [*n* = 19], judo [*n* = 35], kung-fu [*n* = 41], baseball [*n* = 13], tennis [*n* = 15], and track and field [*n* = 11]). Goalkeepers were not included due to particularities in biomechanics compared to other soccer players. The definition of organized sport was based on the presence of training periodization, supervision by a head coach, and participation in regional/national tournaments.

The minimum sample size of 43 adolescents was estimated based on the expected relationship between whole-body BMD and weekly volume of training (*r* = 0.419) [25], considering a statistical power of 80% and significance level of 5% (Z = 1.96). The multivariate model proposed considered four covariates (age, APHV, body fat, and type of sport) and lean soft tissue as mediator. Thus, the minimum sample size was multiplied by the number of covariates and the mediation variable in the model (43 × 5 = 215).

All adolescents reported the number of days per week they trained, as well as how much time per day (h) they spent training (data confirmed by the coach). The weekly volume of training (h/wk) was estimated by the researchers by multiplying time spent training per day x number of days per week. Moreover, in order to be able to take part in this study, the following inclusion criteria were adopted: (I) practice of only one sport; (II) the legal guardian signed the informed consent form.

### Somatic maturation

Body mass (kg) was measured using an electronic scale (Filizola PL 150 model; Filizola Ltds., Brazil). Height (cm) was measured using a stadiometer (Sanny model; American Medical of Brazil Ltda., Brazil). The measures were taken by a qualified technician during a visit the adolescents made to the laboratory. Biological maturation was estimated by the peak of height velocity (PHV) using mathematical models based on anthropometric measures [26]. These equations present the time (in years) remaining (negative values) or past (positive) to PHV, which is an important biological event that follows the human maturation process. Subsequently, the PHV was subtracted from the chronological age, giving the age of peak height velocity (APHV).

$$\mathrm{Years\, from\,age\,of\,PHV\,for\,males}= -8.128741 + [0.0070346^* (Age^* Sitting Height)]$$

### Body composition

The aBMD (g/cm²), fat mass (kg), and lean soft tissue (LST, in kg) were assessed by dual-energy x-ray absorptiometry (DXA; Lunar DPX-NT; General Electric Healthcare, Little Chalfont, Buckinghamshire, United Kingdom) using GE Medical System Lunar software (version 4.7). The precision of the device was tested through the measurements of 30 participants assessed at two moments, resulting in a coefficient of variation (CV) = 0.66% (in whole body aBMD analysis, subjects not included in this sample, as stated in previous studies) [12, 27]. On every day of measurement, a researcher checked the quality status of the scanner before performing the evaluation. The participants wore light clothes and took off any metal accessories (e.g., rings, necklaces, and ear rings) [12, 28]. The measurement was performed in the supine position for approximately 15 min [12, 28]. The radiation dose applied is lower than 0.05mrem [29] with potentially minimal harm to the adolescent’s health.

A single whole-body DXA assessment was performed. Region of interest (ROIs) analysis of aBMD in the upper limbs, lower limbs, spine, and whole body occurred offline after the scans, using the DXA software, following the General Electric Health recommendations and previous studies based [27, 28] on the anatomical points:

- Upper limbs ROI: measured considering the position of the line passing through the upper edge of the acromial extremity of the clavicle. The medial boundary of the ROI (separation of the trunk) was made in the midline of the glenohumeral joint and the lateral boundary defined comprising all soft tissues (the same for right and left sides). [28, 30].
- Lower limbs ROI: Lower limbs: measured considering the position of the line passing through the lower edge of the ischium. The lateral and medial lines defined comprised all soft tissues (the same for right and left sides).[28, 30].
- Spine ROI: Measured from the posterior-superior edge of the iliac crest (L4/L5 level) to the lower edge of the chin. The lateral cut positioned as close as possible to the spine (right and left). [28, 30].

### Statistical Analysis

The descriptive analysis is presented using mean, standard deviation, maximum and minimum, and 95% confidence interval (CI95%). The Kolmogorov-Smirnov was used to test the distribution of the data. Partial correlation was used to assess the correlation between the independent variable (training volume), the mediator (LST), and the dependent variable (aBMD) in different segments (upper limbs, lower limbs, spine, and whole body). The model was adjusted by age, height, APHV, body fat, and the sport the adolescent was engaged in (categorical variable).

**Fig. 1:**
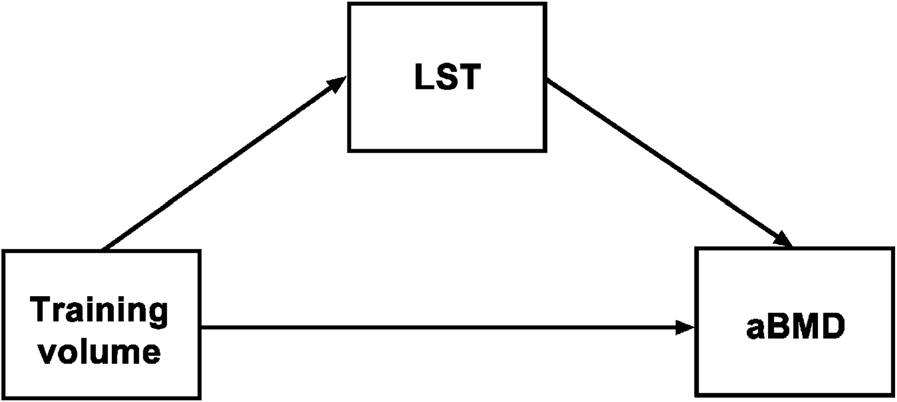
Theoretical model of the association between training volume, areal bone mineral density (aBMD), and lean soft tissue (LST) as a mediator

To investigate the role of LST in the association between training volume and aBMD, a mediation analysis was performed, using the KHB method [31]. In this method, the total effect of the association (in this case, training volume, and aBMD) is decomposed into direct effect (effect of training volume on aBMD adjusted by LST) and indirect effect (i.e., the mediation effect). In other words, we isolate the indirect effect through testing the difference between the total effect (model without the mediator) and the direct effect (model with the mediator) [23]. Furthermore, it is possible to obtain the size of the association which is explained by the mediator variable (mediation in percentage values [%]). A different model was estimated for each outcome. The covariates were chosen by assessing the literature and were included in the model after performing a multicollinearity test. Aiming to reduce the potential role of unobserved/unmeasured confounders, the adjustment variables were age, APHV, fat mass, and the sport [32, 33]. The theoretical model of the mediation analysis is described in Fig. 1. All analyses were performed using STATA 15.1 software and the level of significance was set at *p* < 0.05.

## Results

The final sample was composed of 299 male adolescents, aged between 10 and 18 years (10 years [*n* = 5]; 11 years [*n* = 29]; 12 years [*n* = 37]; 13 years [n = 54]; 14 years [*n* = 49]; 15 years [*n* = 15]; 16 years [*n* = 41]; 17 years [*n* = 33]), with a mean age of 14.1 ± 1.8 years, and APHV of 13.4 ± 0.5 years. All descriptive data are presented in Table 1.

**Table 1:** Characteristics of the sample (*n* = 299)

Variables	Mean (SD)	Minimum to maximum	
Age (years)	14.1 (1.8)	10.1 to 17.7	
Body mass (Kg)	62.3 (15.3)	31.6 to 137.3	
Height (cm)	170.0 (12.2)	136.1 to 196.5	
FM (kg)	11.3 (8.9)	2.1 to 71.4	
FM (%)	18.0 (10.4)	1.5 to 42.8	
LST (kg)	47.3 (11.2)	24.4 to 77.1	
PHV (years)	0.74(1.58)	-2.55 to 3.56	
APHV (years)	13.4 (0.5)	12.02 to 14.98	
aBMD (DXA)			
Upper limbs (g/cm²)	0.827 (0.141)	0.580 to 1.241	
Lower limbs (g/cm²)	1.327 (0.200)	0.835 to 1.878	
Spine (g/cm²)	1.049 (0.167)	0.674 to 1.536	
Whole body (g/cm²)	1.163 (0.142)	0.847 to 1.524	
Previous time of practice (month)	53.8 (42.8)	1 to 153	
Training frequency (days)	3.9 (1.5)	1 to 7	
Training volume (h.wk)	10.6 (6.1)	1.0 to 30.0	

*SD* standard deviation; *FM * fat mass; *LST *lean soft tissue; *PHV *peak of height velocity; *APHV *age at peak of height velocity; *aBMD *areal bone mineral density

Partial correlations were tested between dependent, mediation, and independent variables (training volume, LST, and aBMD of all segments). There was a positive and significant correlation between training volume and aBMD in upper limbs (*r* = 0.131), lower limbs (*r* = 0.334), spine (*r* = 0.220), and whole body (*r *= 0.268). Similar results were found in the analysis of the relationship between LST and aBMD of all segments: upper limbs (*r* = 0.448), lower limbs (*r* = 0.507), spine (*r* = 0.451), and whole body (*r* = 0.548). In addition, LST and training volume were positively correlated (*r* = 0.156) (Table 2).

**Table 2:** Partial correlation between independent, mediator, and dependent variables separated by sites (*n* = 299)

Variables	Training volume		LST	
	r	*p*-value	r	*p*-value
Boys (*n* = 299)				
Training volume (h.wk)	1.000	-	0.156	0.007
LST (kg)	0.156	0.007	1.000	-
Upper limbs (g/cm²)	0.131	0.024	0.448	< 0.001
Lower limbs (g/cm²)	0.334	< 0.001	0.507	< 0.001
Spine (g/cm²)	0.220	< 0.001	0.451	< 0.001
Whole body (g/cm²)	0.268	< 0.001	0.548	< 0.001

**Note**: model adjusted for age, APHV, fat mass, and modality; *p* < 0.05

In the mediation models, LST indirectly influenced the association between training volume and aBMD in upper limbs (β = 0.00142[0.00053 to 0.00231]; 58.37% mediated), lower limbs (β = 0.00191[0.00074 to 0.00307]; 25.32% mediated), spine (β = 0.00149 [0.00054 to 0.00244]; 33.55% mediated), and whole body (β = 0.00158 [0.00063 to 0.00253]; 33.96% mediated). However, interestingly, the direct effect of volume of training on aBMD remained significant in all body sites except for upper limbs (Table 3).

**Table 3:** Mediation role of lean soft tissue on the association between training volume and BMD (*n* = 299).

Mediator	aBMD (g/cm²)	Total effect	*p*-value	Direct effect	*p*-value	Indirect effect	*p*-value	%Mediated	R²
Boys (*n* = 299)	Upper limbs	0.00243	0.009	0.00101	0.286	0.00142	0.002	58.37%	0.64
	Lower limbs	0.00753	< 0.001	0.00562	< 0.001	0.00191	0.001	25.32%	0.78
	Spine	0.00445	0.001	0.00296	0.007	0.00149	0.002	33.55%	0.67
	Whole body	0.00465	< 0.001	0.00307	< 0.001	0.00158	0.001	33.96%	0.75

Note: model adjusted for age, fat mass, age at peak of height velocity, and modality; *LST *lean soft tissue; *aBMD *areal bone mineral density. % mediation was only estimated for a significant indirect effect with a significant total effect.

## Discussion

This cross-sectional study aimed to identity, for the first time, the mediating effect of body LST on the association between training volume and aBMD in male adolescents engaged in impact sports. Our main finding indicates that LST partially mediated the association between training volume and aBMD in male adolescents engaged in sports. Furthermore, the mediation of LST in the association was higher in the upper limbs aBMD (~ 58%) than in the other aBMD sites (25–35%), without a direct association between volume of training and aBMD in this body segment.

Our results corroborate that sports participation seems able to affect aBMD directly (through mechanical impacts) and/or indirectly (through lean soft tissue). This occurs because bone tissue is responsive to demands imposed on its structure [9]. When stresses caused in the bone matrix exceed a modeling threshold, bone starts remodeling its structure to remain strong. In this scenario, muscle contractions are greatly responsible for causing the loading that exceeds the bone modeling threshold, stimulating the bone remodeling process [9]. Furthermore, muscular tissue is a potential secretor of insulin-like growth factor 1 (IGF-I), a hormone that acts on the GH-IGF-I axis and stimulates bone tissue growth, assuming a key role in the interaction of these tissues [34]. In fact, increased LST and muscular fitness have been linked to satisfactory bone tissue parameters in other studies [15, 16, 35, 36].

In the present study, when LST was included in the statistical model, the direct association between training volume and upper limbs aBMD lost significance, with only the indirect pathway remaining significant. In addition, LST mediated nearly 58% percent of the association between training volume and upper limbs aBMD, while mediating 25% of the association with lower limbs aBMD (similar percentages in other body segments). Although we did not measure the amount or intensity of the impact loading performed by our athletes, and considering the modalities observed, it seems reasonable to infer that these considerable differences in the percentage of LST mediation in the upper limbs compared to other body regions can be explained by mechanical loading generated during the sport participation [37].

The lower limbs followed by the spine body segments are directly affected by the mechanical loading generated during movements performed in sports, which generate direct matrix deformation, as well as pressure and fluid drag force, among others, in bone tissue [37]. In the present study, when LST was included in the statistical model, the direct association between training volume and upper limbs aBMD lost significance, with only the indirect pathway remaining significant. In addition, LST mediated nearly 58% percent of the association between training volume and upper limbs aBMD, while mediating 25% of the association with lower limbs aBMD (similar percentages to other body segments). In accordance with our study, the results of Zymbal et al. [23] suggest that the effect of muscle on bone tissue can be attenuated in boys, as their sample was widely exposed to more vigorous physical activity (more impact load forces) [38]. Considering that participation in impact sports frequently involves this intensity of physical activity, muscle mass may in fact have less participation in places where there is a greater impact on the ground, justifying our findings.

The present study demonstrates that training volume is associated with aBMD improvement [11, 14, 39] and, thus, prescribing sport training models based on volume could be a valuable strategy to improve bone parameters. Varley et al. (2017) observed that increasing training volume in the training program of male adolescent soccer players also promoted positive bone adaptations. However, it should be pointed out that the mechanism behind these benefits can vary across different sports, as well as being affected by sex [21, 22]. Regarding practical applications, coaches training young athletes who are interested in improving the bone health of these adolescents, should aim to develop good quality LST through the training volume.

The present study has some limitations. First, the cross-sectional design prevents us from analyzing any causal relationship, yet does enable a significant sample number (*n* = 299). Furthermore, we obtained our sample by convenience and the number of adolescents per group did not allow analysis by each sport group separately. Second, there are some potential unobserved/unmeasured confounders that we could not consider in our analysis, for instance, uncontrolled nutritional aspects such as calcium and vitamin D intake [40] that may be associated with mediator and outcome variables. Third, the LST was not considered in specific body segments, only the whole body LST. However, the aBMD variable (g/cm²) was obtained through total and specific body measurements as well as being measured using equipment considered gold standard. Fourth, despite the use of anatomical points previously used in the literature to define regions of interest (ROIs), the regional aBMD from the whole-body assessment is less accurate than regional assessments. Lastly, the study analyzed only somatic maturation, without indicators of sexual maturation (Tanner for example), as well as information on the socioeconomic status and physical fitness of the sample.

## Conclusions

Higher training volume is associated with higher aBMD in different body segments. These associations are influenced by LST, mainly in the upper limbs. In addition, the influence of LST on aBMD seems to be lower where the body segment is more exposed to mechanical loading during sports practice. Interventions aiming to enhance aBMD should also consider LST as an important variable to be managed. Finally, future studies with large sample sizes are encouraged in order to understand the mediating effect of lean soft tissue on this association in each sport specifically.

## Data Availability

The datasets generated and/or analyzed during the current study are not publicly available due it contains private information from medical records but are available from the corresponding author on reasonable request.
